# Species-specific gill’s microbiome of eight crab species with different breathing adaptations

**DOI:** 10.1038/s41598-023-48308-w

**Published:** 2023-11-29

**Authors:** Giovanni Bacci, Niccolò Meriggi, Christine L. Y. Cheng, Ka Hei Ng, Alessio Iannucci, Alessio Mengoni, Duccio Cavalieri, Stefano Cannicci, Sara Fratini

**Affiliations:** 1 Department of Biology, University of Florence, 50019 Sesto Fiorentino, Italy; 2 The Swire Institute of Marine Science, The University of Hong Kong, Hong Kong, Hong Kong SAR People’s Republic of China; 3 State Key Laboratory of Marine Pollution, City University of Hong Kong, Hong Kong, Hong Kong SAR People’s Republic of China; 4 NBFC, National Biodiversity Future Center, 90133 Palermo, Italy

**Keywords:** Ecology, Evolution, Microbiology

## Abstract

Transitions to physically different environments, such as the water-to-land transition, proved to be the main drivers of relevant evolutionary events. Brachyuran crabs evolved remarkable morphological, behavioral, and physiological adaptations to terrestrial life. Terrestrial species evolved new respiratory structures devoted to replace or support the gills, a multifunctional organ devoted to gas exchanges, ion-regulation and nitrogen excretion. It was hypothesized that microorganisms associated with respiratory apparatus could have facilitated the processes of osmoregulation, respiration, and elimination of metabolites along this evolutionary transition. To test if crab species with different breathing adaptations may host similar microbial communities on their gills, we performed a comparative targeted-metagenomic analysis, selecting two marine and six terrestrial crabs belonging to different families and characterised by different breathing adaptations. We analysed anterior and posterior gills separately according to their different and specific roles. Regardless of their terrestrial or marine adaptations, microbial assemblages were strongly species-specific indicating a non-random association between the host and its microbiome. Significant differences were found in only two terrestrial species when considering posterior vs. anterior gills, without any association with species-specific respiratory adaptations. Our results suggest that all the selected species are strongly adapted to the ecological niche and specific micro-habitat they colonise.

## Introduction

How organisms adapt to their environment is a central question in evolutionary biology. Following—and beyond the criticism of—the hologenome theory of evolution^1^, any multicellular organism evolves with and aided by its interaction with microorganisms, which can perform a plethora of metabolic processes that are absent in their host^2^. Ecological transitions and niche shifts proved to be at the core of many speciation events^3^. This is particularly evident when we consider transitions through physically different environments. These evolutionary pathways are characterised by profound morphological, physiological, behavioural, and reproductive adaptations, which often converge towards similar structures and metabolic pathways, even in phylogenetically distant species^4^.

In metazoans, the water-to-land transition is probably the most dramatic of such evolutionary pathways and occurred multiple times across and within most phyla. Among land adapted phyla, Arthropoda contribute the most to species diversity, with insects and arachnids representing the most successful example of terrestrial adaptation in terms of biomass, functions, and biological diversity^5^. Within the crustaceans, however, we can also find many examples of semi-terrestrial and terrestrial forms, mostly represented by Amphipoda, Isopoda, and Decapoda, which include the largest extant terrestrial arthropod, the coconut crab *Birgus latro*^6^.

Among Decapoda, the true crabs (infraorder Brachyura) are of marine origin but relatively recently they successfully colonised most of the intertidal, and terrestrial habitats, including deserts^7^. The colonization of intertidal and terrestrial environments independently happened many times through two distinct pathways. Some taxa conquered the terrestrial habitats via freshwater systems, whereas other taxa colonised the land from marine intertidal environments^8^. Giomi et al. pointed out that heat tolerance, increased oxygen availability and energy savings could constitute the proximate causal factors driving the evolution of air-breathing at tropical latitudes.

To succeed in the conquest of the land, crabs underwent a series of physiological, behavioural, and morphological changes (for a review see^9^). These novel adaptations involved locomotion and sensory cues^10^, respiration^11–13^, water and ion balance^14–16^, reproduction and larval development^17,18^, foraging and nutrient assimilation^19^, and excretion of nitrogenous waste^16,20^.

Focusing on respiration, we observe a shift of gills function from primarily gas exchange and ion transport in water-breathing crabs^21^, to ion-regulation and nitrogen excretion in air-breathing crabs^12,21–23^. This evolutionary trend, common to various brachyuran families, was driven by the physical collapse of the gills adapted to aquatic environments that led to a reduction of the functional surface area available for gas exchange^4^. The colonization of intertidal and terrestrial habitats by brachyuran crabs have thus required the evolution of new respiratory organs, to replace or support the gills in gas exchange. Such novel organs are represented by branchiostegal lungs in species of the family Ocypodidae^13,24^ or tympana on the walking legs of crabs in the family Dotillidae^25^. In the semiterrestrial species of the family Sesarmidae (the most common taxa in mangrove forests) the gills still remain the only structure devoted to respiration, but they are supported by morphological adaptations aimed to hold the water in the gill chambers, and to recycle and re-oxygenate such water for oxygen exchange^21^.

In a recent review, Cannicci and collaborators^9^ suggested the involvement of host-microbiome interactions in the transition of crabs to semi-terrestrial and terrestrial environments. In this theoretical framework, they hypothesised that microorganisms associated with gills could have facilitated the processes of osmoregulation, respiration, and elimination of metabolites. To test this hypothesis and understand if different terrestrialisation events converged to a similar taxonomic composition of the gills’ microbiome, we performed a comparative targeted-metagenomic analysis on the gills of two shallow marine, water-breathing swimming crabs (the portunids *Scylla paramamosain* and *Thalamita crenata*) and six intertidal or semi-terrestrial species characterised by different levels of terrestralization and by three different breathing systems. Out of the six selected intertidal or semi-terrestrial species, two were ocypodids (*Tubuca arcuata* and *Ocypode ceratophthalmus*), characterized by branchiostegal lungs for breathing^13,24^; two were dotillids (*Scopimera intermedia* and *Tmethypocoelis ceratophora*), which developed tympana on the legs^25^; and two were sesarmids (*Parasesarma continentale* and *Chiromantes haematocheir*), which evolved the water recirculation system to oxygenate their gill chamber^21^. To better explore the possible variation in specie-specific microbial communities associated to gills, which are multi-functional organs, we analysed separately the gills attached to the anterior and the posterior pereiopods, respectively. It is known for marine water-breathing crabs that the anterior gills are primarily engaged in respiration and excretion^26,27^ while the posterior ones are the primary site of active ion transport^27^. In semiterrestrial and fully terrestrial brachyurans, the same spatial pattern of gills’ specialisation has been found in those species that still use gills to breath. In terrestrial species that evolved branchiostegal lungs to breath, conversely, only the posterior gills remain for active ion regulation^10^. Beyond species-specific microbial assemblages, we expected to find a higher similarity in gills’ microbiomes isolated from ecologically similar, and phylogenetically allied, species, as well as different assemblages on the posterior and anterior gills, respectively.

## Results

**Figure 1:**
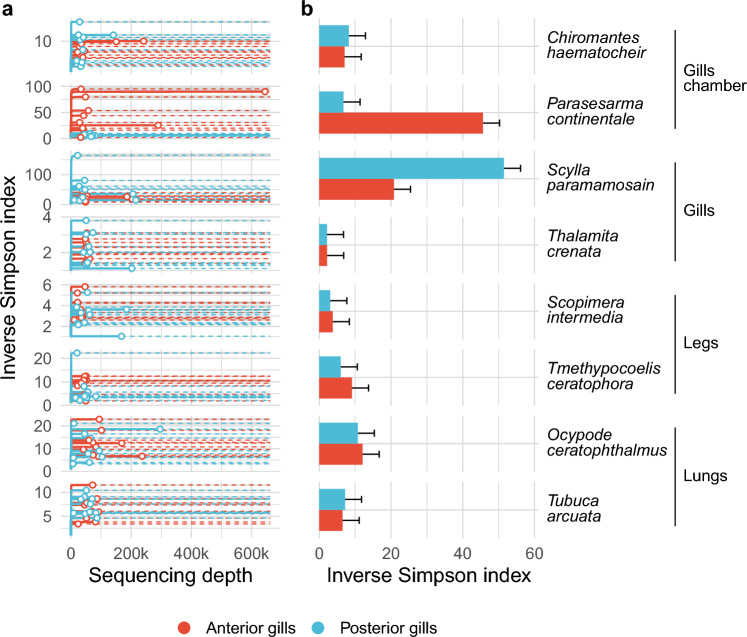
Bacterial diversity against sequencing effort and in different crab species. Bacterial diversity was measured by calculating the inverse Simpson index and reported together with increasing sequencing effort for each sample (panel **a**) and in each crab species (panel **b**). (**a**) Solid lines represent the interpolated biodiversity (sequencing effort lower than the total number of reads assigned to amplicon sequence variants) whereas dotted lines represent extrapolated diversity (sequencing effort higher than the total number of reads assigned to amplicon sequence variants). White points represent the observed diversity in all samples and gray ribbons reports the 95% confidence intervals. (**b**) The average observed inverse Simpson index was reported for each crab species. Standard error on the mean was reported using error bars. Crab species and breathing categories are reported on the right.

**Figure 2:**
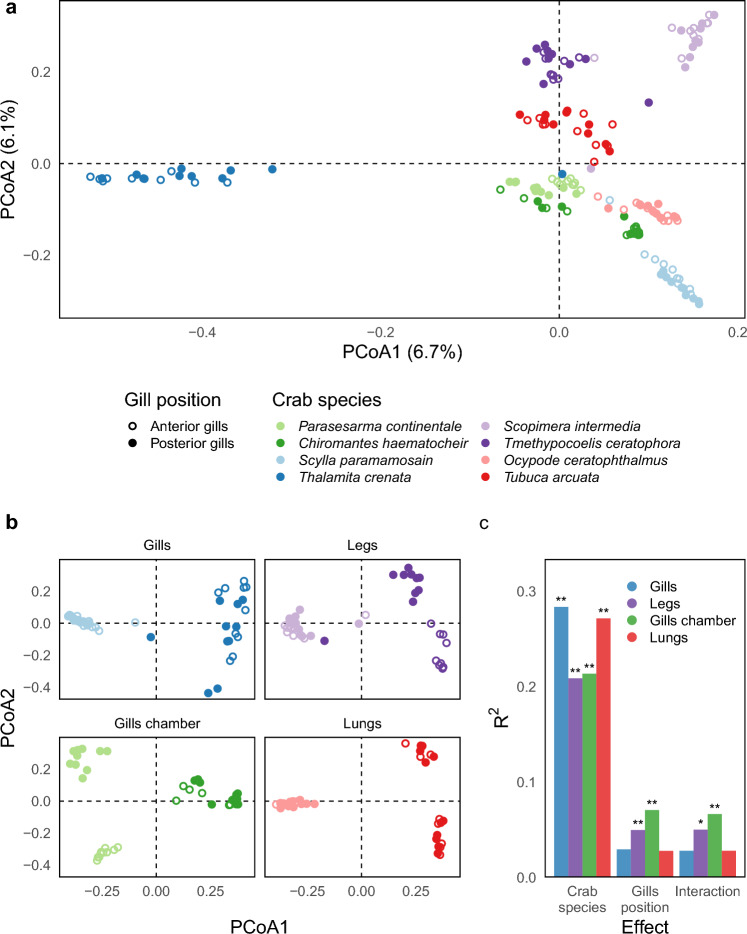
Bacterial diversity distribution across sample groups. Bacterial distribution using Principal Coordinates Analyses (PCoA) based on Bray–Curtis distances inferred from 16S rRNA dataset. Ordination analysis was reported on the entire dataset (**a**) and considering each breathing category separately (**b**). Breathing categories and crab species are reported in the legend following color patterns whereas the anatomical position of the gills (anterior gills and posterior gills) was represented using empty and full circles. R^2^ values from Permutational Analysis of Variance on ordination were reported for the significant contrast inside the tested effects for 16S counts (**c**).

Amplicon sequence variance reconstruction identified 23,130 representative sequences (hereafter called ASVs) with a sequencing depth of 10,360,201 sequences (Fig. S1) for all the eight crab species selected (shown in Table 1). The alpha diversity estimate (inverse Simpson index) suggests that our sequencing effort was enough to reliably explore the microbial diversity of the gills. Indeed, all interpolated rarefaction curves (those obtained using the true alpha diversity detected in each sample before any normalization) reached an asymptote with a difference between the observed and the last extrapolated alpha diversity value between 0 and 1.18. Observed diversity differed both with respect to crab species and to the position of the gills, as reported by the three-way analysis of variance (Table S1). Pairwise contrasts reported the highest diversity in the anterior gills of *Parasesarma continentale* and in the posterior gills of *Scylla paramamosain* with respect to all the other crab species and gills' positions (Figs. 1 and S2). The anterior gills of *S. paramamosain* were significantly different from the anterior and posterior gills of both *Thalamita crenata* and *Scopimera intermedia* (Figs. 1 and S2). The diversity between samples (beta diversity) was also influenced by crab species, breathing category, and gills’ position as reported by the three-way PERMANOVA (Table 2). Since PERMANOVA could be influenced by heteroskedasticity of the tested groups, variance dispersion was assessed by a dispersion test (see Materials and Methods section). Although the effect was mainly driven by a few significant contrasts only (Tukey HSD test: 8 on 155 total combinations, corresponding to ~ 5% of the tested contrasts, Table S2), six contrasts reported a significant effect for the third order interaction, one for species within breathing category, and one for breathing category alone (Table S2). Bacterial distribution was additionally reported using principal coordinates analysis, which confirmed a clear separation according to the breathing category (Fig. 2a,c), as well as the crab species (Fig. 2b). A sharp separation of bacterial communities among the anterior and posterior gills was detected only for two species, namely *Parasesarma continentale* and *Tmethypocoelis ceratophora* (Fig. 2b).

**Table 1:** Studied species.

Family	Species	Breathing category	Intertidal belt	Microhabitat
Portunidae	*Scylla paramamosain*	Gills	Subtidal	Shallow waters on muddy-sandy substrata
	*Thalamita crenata*	Gills	Subtidal	Shallow water on muddy-sandy substrata
Ocypodidae	*Ocypode ceratophthalmus*	Lungs	High intertidal belt	Sandy beaches
	*Tubuca arcuata*	Lungs	Mid intertidal	Mangrove forests on muddy substrata
Dotillidae	*Scopimera intermedia*	Legs	Low intertidal	In front of the mangroves, on muddy-sandy substrata
	*Tmethypocoelis ceratophora*	Legs	Low intertidal	Seaward mangrove belt on muddy-sandy substrata
Sesarmidae	*Parasesarma continentale (*= *bidens)*	Gill chamber	Throughout the intertidal zone	Mangrove forests on muddy substrata
	*Chiromantes haematocheir*	Gill chamber	Supratidal	Landward mangrove belt and riverine forests

List of species included in the study with details relating to breathing category and ecological niche.

**Table 2:** Permutational multivariate analysis of variance (PERMANOVA) on Bray–Curtis diversity index.

Effect	DF	SS	R^2^	F	*P*
Breathing category	3	11.917	0.161	13.790	< 0.001
Gills position	1	0.719	0.01	2.496	< 0.001
Breathing category × Gills position	3	2.074	0.028	2.4	< 0.001
Breathing category × Species	4	15.013	0.203	13.029	< 0.001
Breathing category × Gills position × Species	4	2.724	0.037	2.364	< 0.001
Residual	144	41.481	0.561		

The effect of breathing category, gills’ position, and crab species (Species) on bacterial diversity expressed using the Bray–Curtis index was assessed with a permutation test based on 1,000 permutations. Second and third order interaction were reported using an “x”. Since crab species belonged to different breathing categories (namely two species for each of the four systems included in the work), we explicitly tested the effect of species within breathing category without including the main effect for crab species.

Columns represent: Effect, The factor tested; DF, Degrees of freedom; SS, Sum of squares; R^2^, Percentage of variance explained; F, F-statistic; P, *P* value.

**Figure 3:**
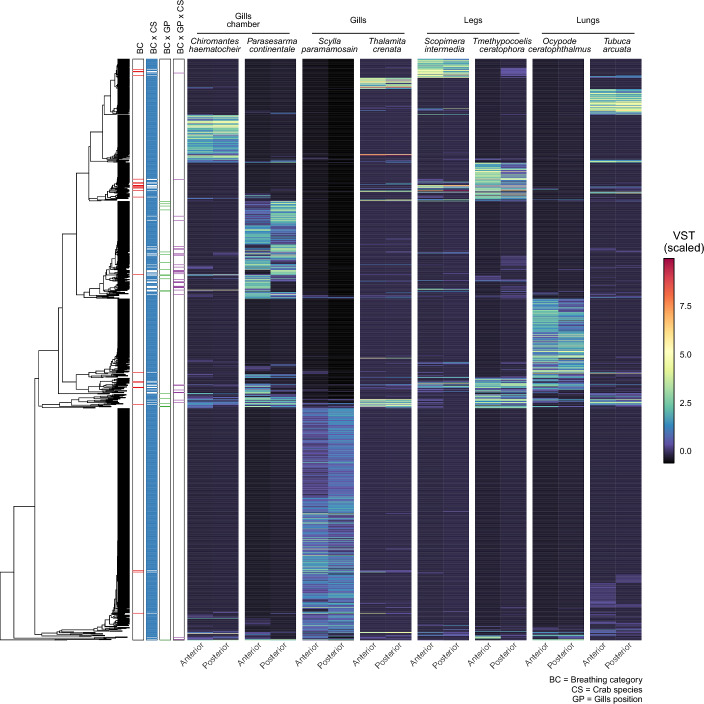
Amplicon sequence variants (ASVs) abundance and distribution. The abundance of ASVs significantly impacted by at least one of the factors tested was reported after variance stabilizing transformation (VST) and standardization to zero mean and unit variance. Transformed abundance values were used to cluster ASVs following Kendall’s correlation coefficient and results were plotted on the left. Factors tested were reported next to the heatmap to highlight significant factors (labels reported on top). Axis text was reported using the same notation described in Fig. 1. Tested factors were abbreviated as follows: *BC* Breathing category, *CS* Crab species, *GP* Gills’ position. Interactions were reported using a “x” between the two (or three) factors tested.

Likelihood-ratio test identified a total of 1037 ASVs significantly influenced by at least one of the tested factors (breathing category, crab species, gills’ position or the interactions between them). The vast majority of the selected ASVs (1022) was affected by one main factor only or by the interaction factor, whereas 15 ASVs were significantly affected by two factors simultaneously (a total of 1052 distinct effects were selected, Table S3). As reported in Table S3, crab species was the dominant factor influencing the bacterial communities of gills (973 of ASVs), followed by gills’ position (55 ASVs) and breathing category (24 ASVs). Hierarchical clustering based on Kendall’s distance (see Material and Method section) on selected ASVs showed a strong effect of crab species while producing a few clusters related to the crab’s breathing category (Fig. 3). This effect was confirmed by the upset plot reported in Fig. S3. The unique microbiome—composed of ASVs detected only on the gills of a single crab species—included the vast majority of the ASVs reconstructed, with *S. paramamosain* being the crab species with the largest number of sequence variants (11,595 ASVs) as well as the one with the largest unique microbiome (10,562 unique ASVs representing 91% of its microbiome). Except for the unique microbiota, the ASVs shared between two different crab species were the most abundant ones (namely, intersections between two sets in the upset representation, Fig. S3), but crab species belonging to the same respiration category seem to share a lower number of AVSs than the others. This effect was mainly driven by *S. paramamosain* which was present eight times on 23 intersections of two or more species including more than 20 ASVs.

Linear discriminant analysis Effect Size (LefSe) identified 2,008 bacterial taxa correlated to (at least) one of the eight crab species considered in the study. The effect size (called LDA score) for each significant bacterial taxa was reported in Fig. S4 whereas bacterial genera only were reported in Fig. S5. Overall, the analysis detected a total of 149 significant taxa with an LDA score higher than three, that can be interpreted as a difference in abundance of, at least, three orders of magnitude (Tables 3 and S4). Among the eight crab species considered, *S. paramamosain* reported a distinctive microbiota with a greater number of taxa, which include 29 different genera, selectively enriched only in this crab species (Figs. S4, S5, and Table S4). Another characteristic species was *C. haematocheir* with seven significant genus-level taxonomic features that were also identified in a previous work of our team (for additional information see^28^). *T. crenata*, *S. intermedia*, *T. ceratophora*, *P. continentale*, *T. arcuata* and *O. ceratophthalmus* showed only a few significant taxonomic observations in respect to the above-mentioned species (Tables 3 and S4, Figs. S4, S5).

**Table 3:** Number of discriminant taxa for each crab species included in the work.

Breathing category	Crab species	Phylum	Class	Order	Family	Genus
Gills (GI)	*Scylla paramamosain*	4	18	23	23	29
	*Thalamita crenata*	1	1	1	2	3
Legs (LE)	*Scopimera intermedia*	0	0	0	0	3
	*Tmethypocoelis ceratophora*	0	0	0	0	2
Gills chamber (GC)	*Parasesarma continentale*	1	1	0	1	1
	*Chiromantes haematocheir*	1	3	4	8	7
Lungs (LU)	*Tubuca arcuata*	0	0	1	4	1
	*Ocypode ceratophthalmus*	0	0	1	1	2

Bacterial taxa associated with crab species were selected using LEfSe and reported in the table. Since we tested all taxonomic ranks from Phylum to Genus, only those taxa reporting a significant effect at a specific taxonomic rank were reported discarding higher taxonomic ranks eventually included in the lineage.

## Discussion

In this study, we investigated the microbiome associated with the gills of six semi-terrestrial crabs that evolved novel morphological adaptations to air-breathing, and we compared them to the gills’ microbiome of two truly water-breathing species. In our sampling design, for the first time, we also characterized the microbial communities of the gills of the selected species considering the anatomical and functional separation present between the anterior and the posterior ones. We found specific taxonomic bacterial profiles significantly associated with each crab species, but such profiles were neither associated with the species’ phylogeny nor with their breathing category. This supports the hypothesis that the gill’s microbiota in brachyuran crabs undergoes a significant selection process at species level, hence suggesting a strict microbiome-host association. We found a significant difference in microbiome associated with the posterior vs. the anterior gills in two species, *Parasesarma continentale* and *Tmethypocoelis ceratophora*, but not in other species belonging to the same families. Possible explanations of these results should consider the fact that all the selected species represent transitional evolutionary steps from sea to land and could show peculiar physiological and ecological features related to the ecological niche and specific micro-habitat they colonise.

The transition from the aquatic to the terrestrial environment requests remarkable physiological, anatomical, and behavioral changes and poses relevant challenges related to respiration, osmoregulation and waste excretion^4^. In marine crabs, all these functions involve the gills, at various levels, while in terrestrial species these organs are primarily involved in ion transport and waste excretion, and new anatomical structures have been evolved for respiration. We recorded a strong specificity of bacterial communities associated with the gills of the eight studied species, regardless of their terrestrial or marine adaptations. This clearly indicates a non-random association of hosts and bacteria that can be explained by a species-specific selection. Species that evolved the same respiratory structures (i.e., lungs, tympana, and a water recirculation system on the branchiostegites) and belong to the same brachyuran family do not cluster together in terms of associated bacterial communities but present a species-specific microbiome on their gills. These results do not support our working hypothesis that species sharing the same respiratory physiology would have had more similar taxonomies on their microbiota and suggest that the gill-associated microbiota could be strongly selected by the host. Consequently, we cannot exclude *a priori* that the gill-associated microbiota may indeed have a role in host’s organ physiology. The different species-specific microbiota found on the gills of hosts under similar ecological pressures (as our terrestrial crabs) might still have similar metabolic functions and trophic interaction with the hosts, despite their different taxonomy^29^, to help coping with gills’ gas exchange functions, as shown in several systems^30,31^.

In a recent paper, Fusi et al. (2023) described bacterial communities associated with the gills of five species of fiddler crab^32^. Interestingly, the authors detected an actinobacterial group (namely *Ilumatobacter*) that is potentially able to convert ammonia into amino acids, and hypothesized its role in helping eliminate toxic sulphur compounds and carbon monoxide. In our study, *Ilumatobacter* was detected as one of the discriminant taxa characterizing the gills’ microbiome of the dotillid *Scopimera intermedia*, in line with our hypothesis that gills’ microbiota may help terrestrial crabs in their physiological and metabolic adaptations to live out of the water.

Considering the phylogeny of the species studied, our results reflect the findings of Boscaro et al.^33^, who did not detect signals of phylosymbiosis studying 1000 microscopic marine invertebrates from 21 phyla. Phylosymbiosis is characterized by the increasing similarity of microbial community among more closely related species. Other than being the product of host-microbe coevolution, it can also be the result of environmental filtering of certain microbes by the similar phenotypic traits displayed by species that share close phylogeny^34^. In our case, neither the grouping based on breathing structures nor the phylogeny of the animals are able to explain the differences among the microbial community across species.

Apart from *C. haematocheir*, which dwells in terrestrial secondary forests, the intertidal species we selected occupy overlapping tidal zones and can be found in close proximity to each other at our sampling sites. For instance, the two swimming crabs, *S. paramamosain* and *T. crenata*, are actively hunting for prey on the very same mudflats and at the seaward mangrove fringes where the other analysed species are out for feeding when tide recedes. As such, their gills are constantly in close contact with the same tidal water system and probably share a common source of microbiomes. Thus, the above-mentioned species seem to be able to select only particular microbiota from the common pool present in the environment. It is also true, however, that every single species occupies a rather different ecological niche and insists on its own peculiar microhabitat, and this small-scale environmental differentiation can also be called upon for the species-specific differences in microbial communities.

The documented species-specific selection also opens to interesting considerations on the transmission of microbial communities across generations of species with indirect development, which involves the release of planktonic larvae in coastal waters. The possibility of vertical transmission through the parental-offspring pathways appears rather remote especially for our semi-terrestrial and terrestrial species. Thus, the more probable hypothesis should be a pseudo-vertical transmission through indirect contact among adults and juvenile individuals.

The microbial communities found on the crabs’ gills are abundant and rich in many different bacterial taxa: this is not surprising as it is known that the crabs’ gills are coated by many bacteria that cover the entire gill lamellae^35^. Guariglieri et al.^35^ analysed gill-associated bacteria in crabs of the families Grapsidae, Ocypodidae, Portunidae, and Sesarmidae from South Africa, Kenya and Saudi Arabia through microscopy observations and described rod-shaped bacteria and cocci of different sizes, different across the species analysed and distinct from the microbes found in the surrounding environment. Bacterial endosymbionts associated with crabs’ gills have been also reported for two Caribbean mangrove crabs, *Aratus pisonii* (Sesarmidae) and *Minuca rapax* (Ocypodiade), by Béziat et al.^36^. In agreement with Guariglieri et al.^35^, Béziat et al.^36^ observed a dense biofilm of bacteria across the surface of gill lamellae and found differences in microbial communities associated with the two species. Despite being focused on species collected from distant geographic area (i.e., South Africa, Kenya, Saudi Arabia and Guadeloupe), both these studies^35,36^ reported a prevalence of Proteobacteria, Actinobacteria and Bacteroidetes. We found that members of Proteobacteria phylum (in particular those belonging to the class Gammaproteobacteria) are dominant only in the portunid crab *Thalamita crenata*. This may be attributed to the different taxonomic depth used to profiling the microbial communities. Indeed, in the above cited works microbial composition was resolved only at Phylum level, making speculation at higher taxonomic levels (as reported in our work) not directly comparable.

Crustaceans’ gills are known to be multi-functional organs^27^. As osmoconformers, gills in fully marine crustaceans are primarily responsible for respiratory purposes. While maintaining extracellular osmotic balance becomes increasingly challenging in semi terrestrial or terrestrial species, gills decouple from their primary respiratory function as the animals develop new morphological adaptations and various strategies to extract oxygen from air. As such, gills differentiate into an anterior and a posterior functional group, the former mainly devoted to respiratory services and the latter enriched with ionocytes to carry out ionic and osmotic regulatory functions^27^. Only two of our semi-terrestrial species, however, hosted different bacterial communities on the anterior and posterior gills, respectively, namely *Parasesarma continentale* and *Tmethypocoelis ceratophora*. These two species belong to two different breathing categories and the diversity in the microbial profiling associated with the two gills’ portions is not explainable *tout court* as due to the respiratory adaptations. The significant differences found between the microbiota associated with anterior and posterior gills are not family specific as well, since we could not confirm such differences in the other crabs belonging to Sesarmidae and Dotillidae, *Chiromantes haematochier* and *Scopimera intermedia*, respectively. All the four species are bimodal breathing crabs, able to gather oxygen from both water and air, and all of them (except for *C. haematocheir*) are strictly intertidal crabs. Bimodal breathing crabs inhabiting intertidal areas are transitional evolutionary stages in the invasion of the terrestrial habitat by aquatic forms (see^37^). Bimodal breathing species, in fact, simultaneously exchange respiratory gasses with air and water across two distinct respiratory epithelia, with O_2_ uptake occurring preferentially from air and CO_2_ excretion occurring across the gills into the branchial water^37^. Conversely, strictly air breathing, truly terrestrial crabs can excrete CO_2_ into air across the gills and the branchiostegites^37^. The species-specific adaptation of the gills to increasing terrestrialisation of intertidal, bimodal-breathing species considered in our study may be the reason why we detected differences within families, which could encompass species that developed slightly different degrees of adaptation to air-breathing and terrestrial-style osmoregulations. Further studies focused on other semiterrestrial and terrestrial sesarmids and dotillids are needed to confirm if differential microbiota associated to anterior and posterior gills are linked to a progression of physiological adaptation to terrestrial life.

As mentioned above, the fact that the microbiota of each host species remains distinctive when compared to that of all other species suggests the possibility of a strong host-microbiota association. Indeed, apart from few ubiquitous taxa, present across all species, we found specific taxa within each microbial communities that we can assume to be relevant for biological functions and environmental adaptations of the host. Under this working hypothesis, *Methylotenera, Moheibacter* and *Chryseobacterium* genera, found in the gills’ microbiome of *C. haematocheir*, are bacteria involved in the removal of nitrogenous compounds. *Methylotenera* include denitrifying and methanol-digesting bacterial strains that play a role in ammonium excretion^38,39^. The genera *Moheibacter* and *Chryseobacterium* were also described for their denitrifying properties^40,41^. *Moheibacter* and *Methylotenera* have been already isolated from the gills of *C. haematocheir* individuals sampled from different Hong Kong populations, confirming that they are stable residents of the microbiome associated to the gills of this species^28^.

The microbial communities associated to various organs of the mud crab *Scylla paramamosain* have been recently described by Zhang et al.^42^. The authors investigated the effect of molting on microbiome and recorded dynamic changes in abundance and composition of microbial communities harbored by the gills and midgut. Molting occurs regularly in crabs to grow and replace old exoskeleton, including gills. The authors described a decrease in bacterial abundance immediately after molting and a gradual recovering during the post molting phase. However, the core microbiota did not change during the whole molt cycle, although some microorganisms disappeared^42^. This suggests that bacterial communities associated with the external organs undergo a drastic selection. However, the occurrence of a stable resident communities associated with the gills of S. *paramamosain*—as confirmed by our study—indicates a non-random selection in favor of taxa that play a role in ecological adaptations of the host.

This study is the first extensive attempt to compare patterns of alpha and beta diversity for microbial communities associated with the anterior and posterior gills of a large number of marine and semi-terrestrial crab species. Our results show that these crabs are an interesting model for the study of holobiont theory of evolution in relation to the transition to land, since we observed a remarkable lack of overlap among the gills’ microbiome associated with each crab species. Ultimately, our results confirm the recent findings that no phylosymbiosis is present in taxa of marine origin. This non-random association of hosts and bacteria indicates a species-specific selection, not correlated to the environment, and is consistent with an evolutionary model of host-microbe association in which at least some components of these microbial communities are specifically selected in favour of ecological and physiological needs of the hosts.

## Methods

### Study species

We selected a total of eight brachyuran crab species based on their physiological and morphological adaptations to gas exchange (Table 1). All our model species are common inhabitants of soft-bottom coastal environments of the Hong Kong SAR territory (PR China) (Table 1). Two of them are confined underwater, in shallow subtidal areas, while the other six are truly intertidal, and even supratidal, species that colonise mangrove and the adjacent secondary forests. For each species, 10 sexually mature adult individuals in intermoult stage were collected along the sheltered bays of Tung Chung and Shui Hau (Lantau Island), and of Pak Tam Chung and Yung Shue O (New Territories). Crabs were immediately frozen and subsequently transported to the laboratories of the School of Biological Sciences (The University of Hong Kong). Anterior and posterior gills were then dissected under sterile conditions, preserved in RNAlater™ Stabilization Solution (Thermo Fisher Scientific) and stored at − 20 °C until DNA extraction. Animal samplings were performed in compliance with local and institutional regulations.

The two shallow subtidal species were the swimming crabs *Scylla paramamosain* and *Thalamita crenata* (Portunidae), which live in sympatry on the intertidal platforms extending in front of the mangroves. They are both generalist predators active at high tide^43,44^. Being swimming crabs, they use their gills to breathe, mainly the anterior ones^45^. Hereafter, we will group these two species within the breathing category “gills”.

Our six intertidal model brachyuran species belong to three families characterized by different breathing adaptations. *Tubuca arcuata* is a fiddler crab that inhabits the intertidal areas sheltered by the canopy of mangrove trees, while the ghost crab *Ocypode ceratophthalmus* digs deep burrows on muddy-sandy beaches^46^. Both species belong to the family Ocypodidae, they are active in air at low tide and evolved a branchiostegal lung, an area of improved blood circulation that lines the branchiostegal chamber where their blood can exchange oxygen with air^11,13^. We categorized these two species within the breathing category “lungs”. Then, we selected two species of the family Dotillidae, *Scopimera intermedia* and *Tmethypocoelis ceratophora*, both colonising muddy-sandy substrata under the most seaward mangrove trees and extending on the platforms lying in front of the forests. Both these species are actively feeding at low tide^47,48^ and, at least partly, use as aerial gas exchange surfaces the membranous disks, known as ‘tympana’, on the meral segments of their walking legs^25,49^. Hereafter, these two species will be referred to within the breathing category “legs”. The last two species we selected belong to the family Sesarmidae and are *Parasesarma continentale* (= *bidens*), the dominant species in Hong Kong mangrove forests^47^, and *Chiromantes haematocheir,* which inhabits areas of coastal secondary forests and pockets of riverine forests^50^. As all Sesarmidae, these two species show a reticulated pattern of setae on their branchiostegites, which they use to recirculate water contained in their gill chambers to maintain a high oxygen level during their aerial activities^21^. In our categorization, these latter species belong to the breathing category “gill chamber”.

### DNA extraction and 16S rRNA (V3–V4) gene amplification and sequencing

Anterior and posterior gills were dissected under sterile conditions, preserved in RNAlater™ Stabilization Solution (Thermo Fisher Scientific) and stored at − 20 °C until DNA extraction. Total DNA extraction was performed by using DNeasy PowerLyzer PowerSoil Kit (QIAGEN) following manufacturer’s instructions. Extracted DNAs were quantified fluorometrically using Qubit dsDNA HS Assay Kit (Thermo Fisher Scientific), then stored at − 20 °C for further 16S metagenomic library preparation.

The bacterial V3–V4 16S rRNA fragments were amplified by using FastStart High Fidelity PCR System (Roche) with the primer pairs 341F (5′-CCTACGGGNGGCWGCAG-3) and 805R (5′-GACTACNVGGGTWTCTAATCC-3′)^51,52^ with overhang Illumina adapters. Each reaction was prepared with 1X FastStart High Fidelity PCR buffer, 1.8 mM MgCl_2_, 0.4 μM of forward and reverse primers, 0.25 mM each deoxynucleoside triphosphate (dNTP), 2.5 μl of template DNA (5–20 ng/μl) and 2,5 U of FastStart High-Fidelity Enzyme.

PCR products were checked through electrophoresis on 1.5% agarose gel and then purified using KAPA Pure Beads (Roche) following the manufacturer’s instructions. The indexing step was performed with 2 × KAPA HiFi HotStart ReadyMix (Roche) using Nextera XT Index Kit V2 (Illumina) in accordance with the Illumina 16S metagenomic library preparation protocol^53^. Indexed PCR products were purified using KAPA Pure Beads (Roche) and their quality check was performed using Agilent 2100 Bioanalyzer (Agilent Technologies) with Agilent DNA 1000 Kit (Agilent Technologies). The subsequent concentration check was performed by using Qubit dsDNA HS Assay Kit (Thermo Fisher Scientific). The barcoded libraries were balanced, pooled at equimolar concentrations, and sequenced on an Illumina MiSeq (PE300) platform at Laboratory of Advanced Genomics, Department of Biology, University of Florence (Italy).

### Amplicon sequence variants reconstruction

Exact amplicon sequence variants (ASVs) were reconstructed from raw sequences by using the DADA2 pipeline (version 1.14.1)^54^. Primers used for PCR amplification (see chapter above) were removed with cutadapt tool version 1.15^55^ in paired-end mode—if a primer was not detected in one of the mates (R1 or R2) the whole sequence was discarded together with its mate to reduce biases due to chimeric PCR amplification. Low quality reads were filtered using the “filterAndTrim” function with an expected error threshold of 2 for both forward and reverse read pairs, namely only reads with more than 2 expected errors were removed. Denoising was performed using the “dada” function after error rate estimation (“learnErrors” function). Denoised reads were merged discarding those with any mismatches and/or an overlap length shorter than 20 bp (“mergePairs” function). Chimeric sequences were removed using the “removeBimeraDenovo” function whereas taxonomical classification was performed using DECIPHER package version 2.14.0 against the latest version of the pre-formatted Silva small-subunit reference database^56^ (SSU version 138 available at: http://www2.decipher.codes/Downloads.html). All variants not classified as Bacteria were removed together with sequences classified as chloroplasts or mitochondria.

### Data analysis

All statistical analyses were performed in the R environment (version 4.1). Diversity analyses were conducted using the vegan R package version 2.5-6^57^.

Bacterial diversity within samples (namely alpha diversity) was measured according to the inverse Simpson index which is defined as the reciprocal of the Simpson index $D$ ($D^{\prime }$). The same index can be defined as a diversity measure with a Hill number of order two^58^ represented with the equation:

$$qD = \left( {\mathop \sum \limits_{i = 1}^{R} p_{i}^{q} } \right)^{{1/\left( {1 - q} \right)}}$$

where $qD$ is the inverse Simpson index, $p_{i}$ is the relative abundance of species $i$ in a given sample, and $\sum\nolimits_{i = 1}^{R} {p_{i}^{2} }$ corresponds to the definition of the Simpson index. This index is particularly useful since it transforms the Simpson concentration–that is by definition the probability of drawing two equal species taken at random from the dataset–into a measure of diversity so that the higher the index, the higher the microbial diversity. We used the “iNEXT” function of the iNEXT R package (version 2.0.20)^59^ to compute interpolated, observed and extrapolated values of alpha diversity with a number of bootstrap equals to two and fixed size for all samples. The iNEXT package calculates interpolated and extrapolated values of diversity as a function of the sample size (called $m$) using the formula reported in the paper from Chao and colleagues^60^. In particular an interpolated diversity estimator ($^{q} \hat{D}\left( m \right)$) is derived for any size $m < n$ where $n$ is the total counts in the sample. Similarly, an extrapolated diversity estimator is derived ($^{q} \hat{D}\left( {m + n} \right)$) for any enlarged sample of size $m + n > n$. Observed microbial diversity is computed by using the diversity formula described above. Confidence intervals were constructed using the bootstrap method and joined to recreate a smooth curve^61^. Generated curves were extended to the maximum sampling size of all samples in order to check if the sequencing effort could be considered enough for the comparison of alpha-diversity across different samples (namely, the inverse Simpson index).

Differences in observed alpha diversity were inspected using a three-way analysis of variance (ANOVA) without including the main effect of crab species. Pairwise contrasts were computed using last-squares means method implemented in the “emmeans” function of the emmeans R package (version 1.7) adjusting resulting p-values with the Benjamini–Hochberg method (also known as false discovery rate or FDR).

The factors considered in the analyses were represented by crab species (referred to as *species*), breathing category (referred to as *breath*) and the anatomical section of gills, i.e. anterior or posterior district (referred to as *gills’ position*). Differences between samples (namely beta diversity) were estimated by using Bray–Curtis index on relative abundance data derived from bacterial counts whereas differences in terms of ASV presence/absence across groups were inspected by using the UpSetR package version 1.4.0^62^ implemented in MicrobiotaProcess package^63^. Data were standardized before beta diversity estimation using the square root of Wisconsin double standardized relative abundance values to uniform samples before comparison (“rcuaten” function of the vegan R package)^64^. Distances were reported using principal coordinate analysis (PcoA, also known as classical multidimensional scaling) implemented in the “cmdscale” function of stats R package. The effect of crab species, breathing systems, and gill portions on bacterial diversity was tested using permutational multivariate analysis of variance (also known as PERMANOVA) implemented in the “adonis2” function of the vegan package. Since different crab species possess different breathing systems (or, in other words, species are implicitly nested into breathing systems), the main effect of species was omitted from our model. To assess the multivariate homogeneity of group dispersions, a beta dispersion test was performed using the “betadisper” function (vegan package). Pairwise differences in dispersion were tested using Tuckey post-hoc test for all groups specified in the model.

Differential abundance analysis was performed using the likelihood-ratio test (LRT test) implemented in the DESeq2 package^65^. Singletons were removed from our data to reduce computational power needed and to dampen the hypothesis that extremely rare species could be considered major drivers of differences in groups. Size factor estimation was performed using the “poscount” method to estimate species geometric means in presence of zeros (“estimateSizeFactors” function of the DESeq2 package). Species dispersion was fitted using local regression (“estimateDispersions” function with “local” fitting method, DESeq2 package). Finally, the LRT test was performed using a nested approach as reported in Fagorzi et al.^66^. For each bacterial species detected, a full model was built by using the breathing category as main effect in combination with second and third order interactions of crab species and gill portions. Terms were then removed one by one starting from highest order terms (interactions) to test their impact on the likelihood of the full model (for additional information see the DESeq2 documentation at http://bioconductor.org/packages/devel/bioc/vignettes/DESeq2/inst/doc/DESeq2.html#likelihood-ratio-test). Bacterial species with at least one significant term were used for clustering analysis. First, species abundance was transformed using the variance stabilizing transformation implemented in the DESeq2 package (“varianceStabilizingTransformation” function). Then values were standardized to unit variance and mean equal to zero using the “scale” function of R base package. A dendrogram was then produced based on the Kendall rank correlation coefficient (τ). Since Kendall’s coefficient ranges between − 1 and 1 (where − 1 means a perfect negative correlation, 1 means a perfect positive correlation, and zero means no correlation), values were transformed into distances (D) according to:

$$D = \left( {\tau - 1} \right)^{2}$$

Distances were then used to perform a hierarchical clustering by unweighted pair group method with arithmetic mean (UPGMA) as implemented in the “hclust” function of the stat R package.

To assess statistically significant features a linear discriminant analysis effect size (LefSe) on relative abundances Table was performed (LDA > 3)^67^. LefSe was conducted by using Galaxy implementation (https://huttenhower.sph.harvard.edu/galaxy/) to identify bacterial changes in the eight crab species considered. LefSe was conducted setting the class vectors considering crab species as class, crab organ as subclass, sample id as subject. Alpha value 0.05 was considered for the factorial Kruskal–Wallis test among classes and for the pairwise Wilcoxon test between subclasses. One-against-all as a strategy for multi-class analysis was conducted. Significant taxonomic features from LDA score (> 3) were graphically depicted in the R environment.

### Supplementary Information

Supplementary Information 1.

Supplementary Information 2.

Supplementary Information 3.

Supplementary Information 4.

## Data Availability

Sequencing data was uploaded to the European Nucleotides Archive (ENA) under project ID: PRJEB51222.
