# Supplementary material for: Species-specific gill’s microbiome of eight crab species with different breathing adaptations
Source: Sci Rep. 2023 Nov 29;13:21033. doi: 10.1038/s41598-023-48308-w (PMC10687215; doi:10.1038/s41598-023-48308-w)
Supplement: Supplementary file 1 — Supplementary Information 1. [file 41598_2023_48308_MOESM1_ESM.pdf]

Supplementary Materials

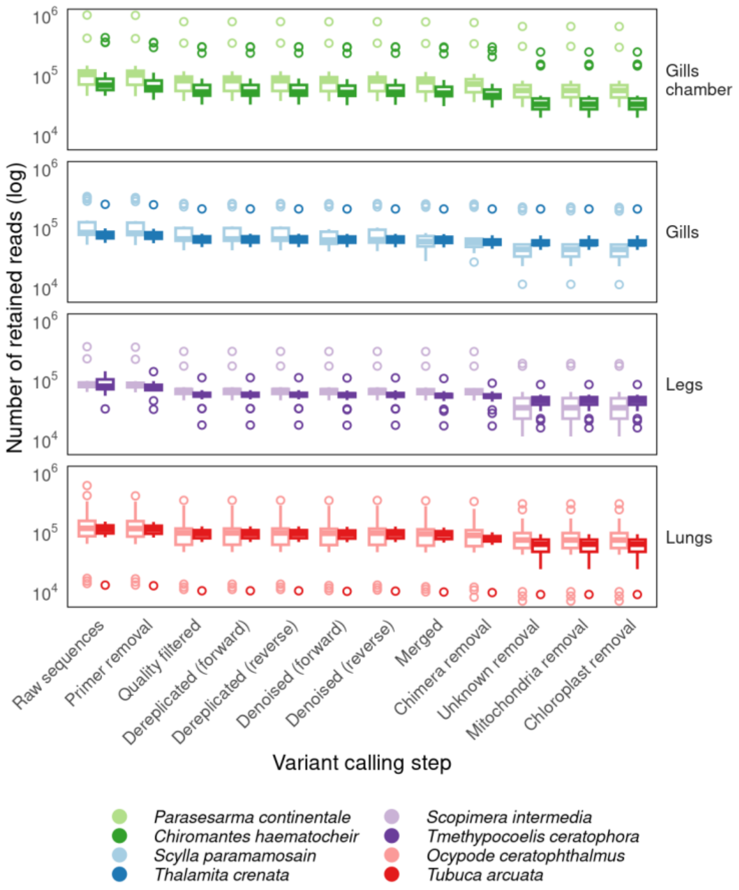

Figure S1. **Retained reads after DADA2 clustering analysis**. Number of reads (y axis; log10 scaled) retained after each step of the DADA2 pipeline (x axis). Results were obtained from 16S rRNA gene amplicons and reported in four different panels based on the crab's breathing category. In each panel the different colors referred to different crab species.

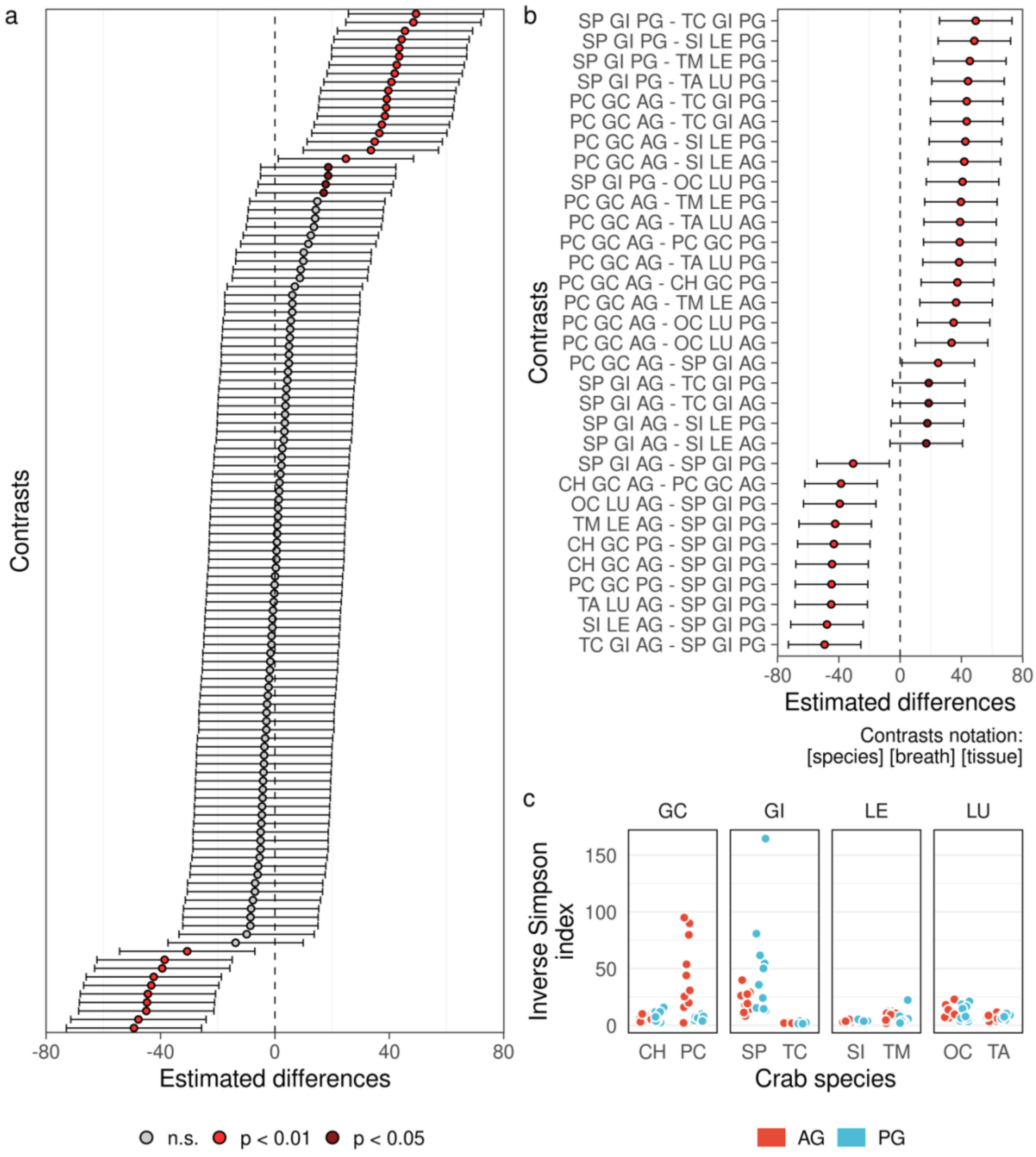

Figure S2. Confidence intervals of pairwise contrasts for alpha diversity. Observed alpha diversity (inverse Simpson index) was compared across multiple groups. The diversity of anterior and posterior gills (AG and PG, respectively) was compared in all the eight crab species included in the work and results were reported in panel a. Significant contrast were reported in panel b following the notation: [crab species] [breathing category] [position of the gill]. The following abbreviations for breathing categories and crab species were used: GI, gills; LE, legs; GC, gills chamber; LU, lungs; SP, *Scylla paramamosain*; TC, *Thalamita crenata*; SI, *Scopimera intermedia*; TM, *Tmethypocoelis ceratophora*; PC, *Parasesarma continentale*; CH, *Chiromantes haematocheir*; OC, *Ocypode ceratophthalmus*; TA, *Tubuca arcuata*. The observed values of inverse Simpson index were reported in panel c.

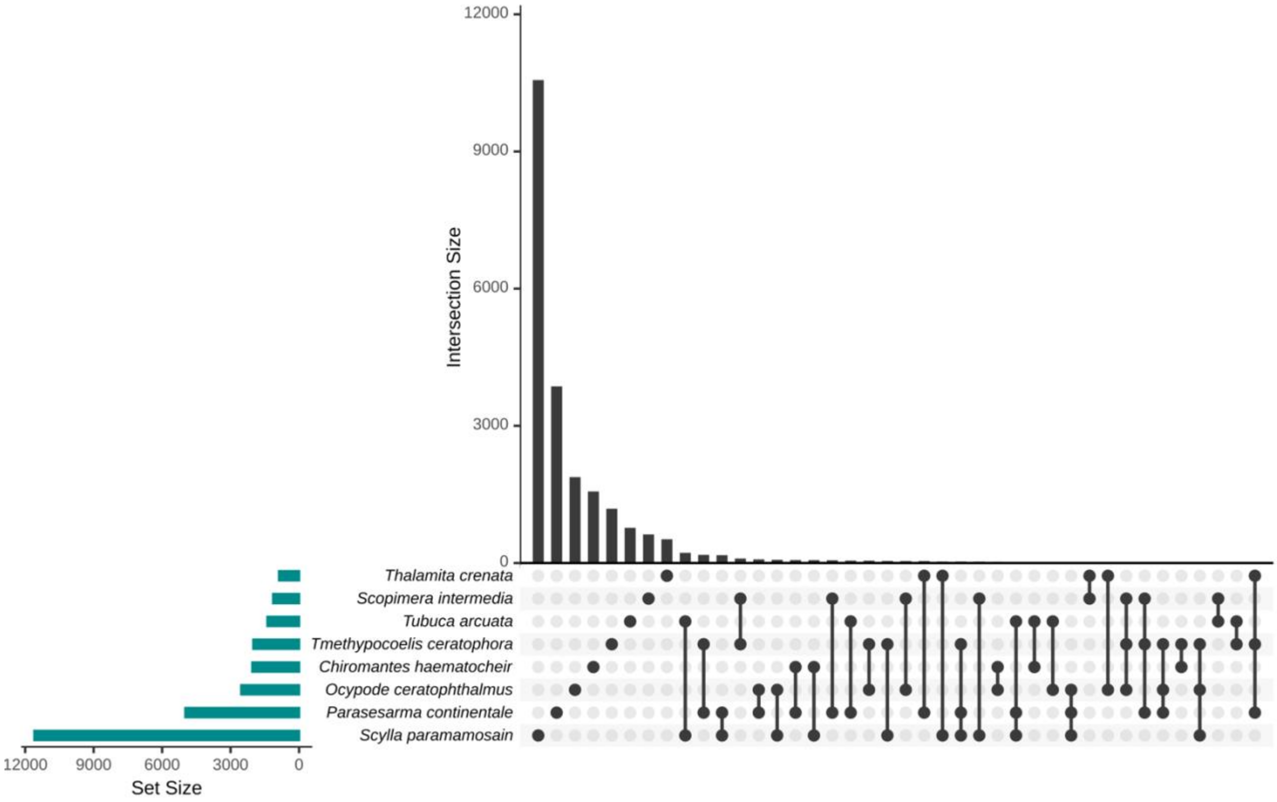

Figure S3: Number of ASVs shared across crab species. The number of ASVs shared between different set intersections was reported following the upset representation. Set intersections were displayed in a matrix layout where each row is a different crab species, and each column corresponds to a different intersection. The number of ASVs in each intersection is reported using bars on top of the intersection considered whereas the total number of ASVs present in the microbiome of a given crab species was reported using horizontal bars (on the left of the matrix layout). Similar to a Venn diagram, intersections are mutually exclusive so that if an ASV is present in a given intersection it is excluded from the others.

Figure S4. Complete taxonomic profiles produced by Linear discriminant analysis effect size (LEfSe) among sample groups. Linear discriminant analysis score (LDA score) obtained after LEfSe analysis reported for the 149 significant abundant features among crab species. LDA score values were reported on x-axis while the detected taxonomic features were reported on y-axis.

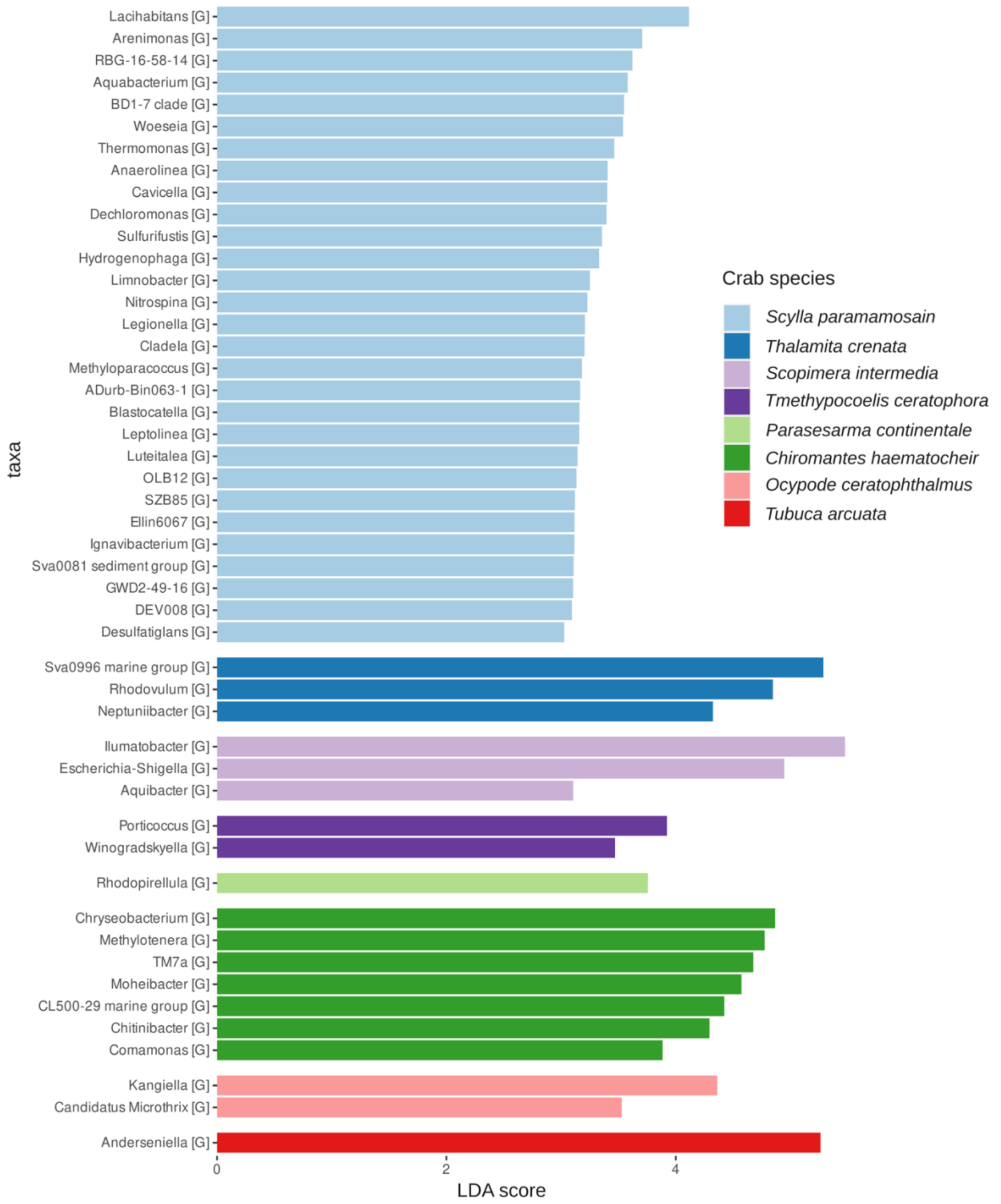

Figure S5. Genus-level taxonomic profiles produced by Linear discriminant analysis effect size (LEfSe) among sample groups. Linear discriminant analysis score (LDA score) obtained after LEfSe analysis reported for the 48 significant abundant features among crab species. LDA score values were reported on x-axis while the detected Genus-level taxonomic features were reported on y-axis.

Table S1: Analysis of variance of observed diversity. Three-way analysis of variance (ANOVA) on observed alpha diversity measured using the inverse Simpson index. Since crab species are implicitly nested into breathing category (a species cannot have more than one category) the main effect of crab species was not included in the model. Effect, term tested; DF, degrees of freedom; SS, sum of squares; Mean^2, mean squared; F, value of F-statistic; P, p-value.

Effect	DF	SS	Mean^2	F	P
Breathing category	3	4983.17	1661.06	7.76	< 0.001
Gills position	1	84.15	84.15	0.39	0.5317
Breathing category x Gills position	3	5852.95	1950.98	9.11	< 0.001
Breathing category x Species	4	15353.30	3838.33	17.93	< 0.001
Breathing category x Gills position x Species	4	6398.27	1599.57	7.47	< 0.001
Residuals	144	30828.35	214.09		

Table S2. Pairwise Tukey HSD beta dispersion. Crab species, breathing categories and gills' position were abbreviated as follows, SP: *Scylla paramamosain*, TC: *Thalamita crenata*, SI: *Scopimera intermedia*, TM: *Tmethypocoelis ceratophora*, PC: *Parsesarma continentale*, CH: *Chiromantes haematocheir*, OC: *Ocypode ceratophthalmus*, TA: *Tubuca arcuata*. GI: gills, GC: gills chamber, LE: legs, LU: lungs. AG: anterior gills, PG: posterior gills. Effect, factor tested; Group.1 and Group.2, the two groups tested (pairwise contrast); diff, differences between the mean distance-to-centroid; lwr and upr, lower and upper 95% confidence interval; p.adj, p-value after Benjamini–Hochberg correction. Significant contrasts were highlighted with an asterisk in the "sign" column.

Table S3. Results of the nested likelihood ratio test (LRT) on ASVs. The number of ASVs reporting (at least) a significant effect was reported along with its percentage value calculated on the total ASVs detected.

Effect	Significant ASVs	Percentage on total ASVs detected
Breathing category	24	0.104
Breathing category x Species	973	4.207
Breathing category x Gills position	19	0.082
Breathing category x Gills position x Species	36	0.156

Table S4. Summary of features obtained from LEfSe analysis reported considering the taxonomic rank. For each taxon, the relative number of observations in each crab species is also reported (n° observation: crab species).
