# Supplementary figures and images for: Species-specific gill’s microbiome of eight crab species with different breathing adaptations
Source: Sci Rep. 2023 Nov 29;13:21033. doi: 10.1038/s41598-023-48308-w (PMC10687215; doi:10.1038/s41598-023-48308-w)

taxa

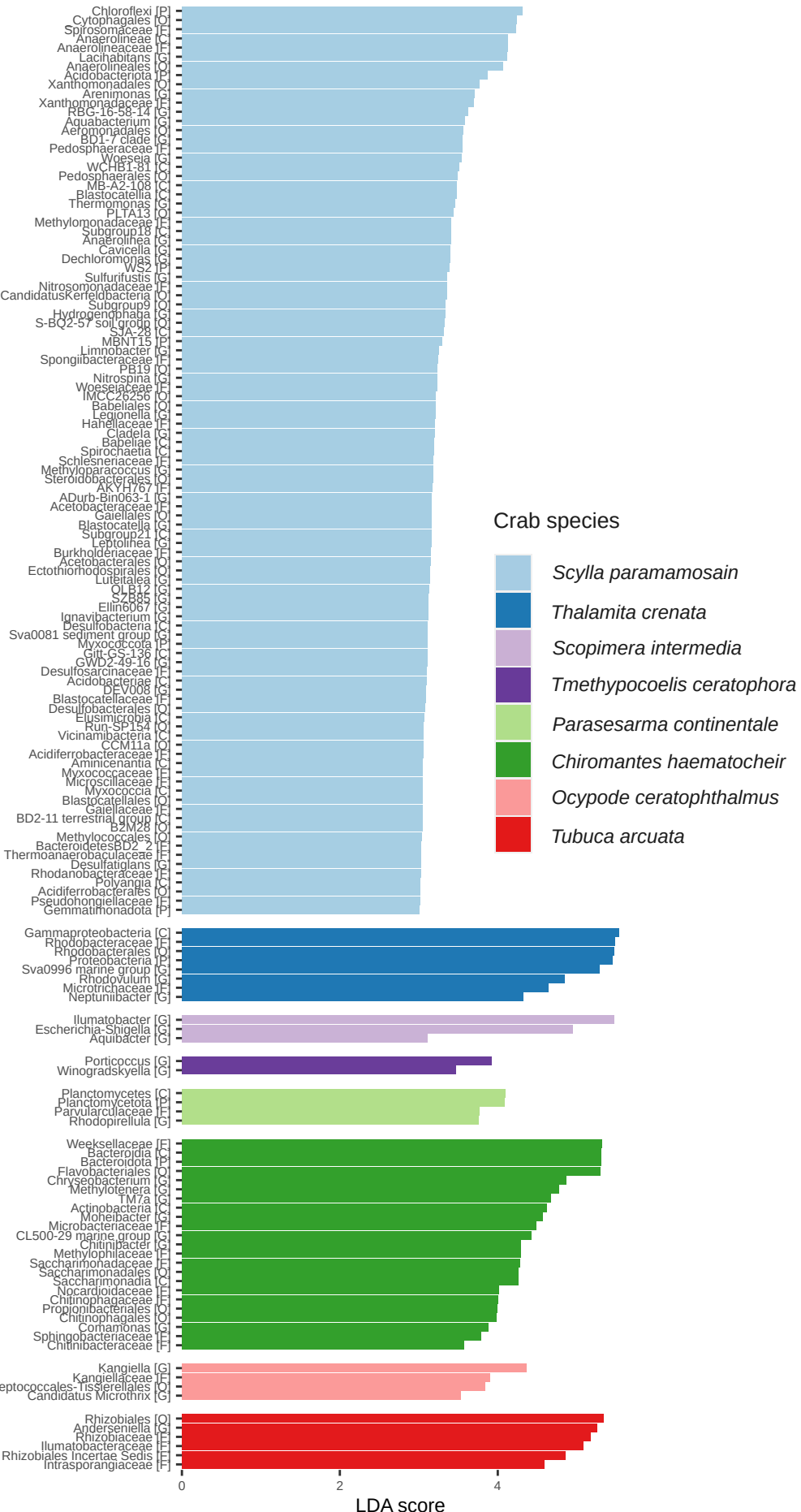

Supplement: Supplementary file 2 — Supplementary Information 2. [file 41598_2023_48308_MOESM2_ESM.pdf]
